# SPECT/CT imaging of inflammation and calcification in human carotid atherosclerosis to identify the plaque at risk of rupture

**DOI:** 10.1007/s12350-021-02745-0

**Published:** 2021-07-27

**Authors:** K. Van der Heiden, H. E. Barrett, E. J. Meester, K. van Gaalen, B. J. Krenning, F. J. Beekman, E. de Blois, J. de Swart, H. J. M. Verhagen, A. van der Lugt, J. P. Norenberg, M. de Jong, M. R. Bernsen, F. J. H. Gijsen

**Affiliations:** 1 Department of Biomedical Engineering, Thorax Center, Erasmus MC, Rotterdam, The Netherlands; 2 Department of Radiology & Nuclear Medicine, Erasmus MC, Rotterdam, The Netherlands; 3 Department of Cardiology, Erasmus MC, Rotterdam, The Netherlands; 4 MiLabs, B.V, Utrecht, The Netherlands; 5 Section Biomedical Imaging, Department Radiation Science & Technology, Delft University of Technology, Delft, The Netherlands; 6 Department of Translational Neuroscience, Brain Centre Rudolf Magnus, University Medical Centre Utrecht, Utrecht, The Netherlands; 7 Department of Vascular Surgery, Erasmus MC, Rotterdam, The Netherlands; 8 Radiopharmaceutical Sciences, University of New Mexico, Albuquerque, NM USA; 9 Applied Molecular Imaging Erasmus Core Facility, Erasmus MC Rotterdam, Rotterdam, The Netherlands

**Keywords:** SPECT/CT, calcification, inflammation, human carotid plaque, vulnerable plaque

## Abstract

**Background:**

Calcification and inflammation are atherosclerotic plaque compositional biomarkers that have both been linked to stroke risk. The aim of this study was to evaluate their co-existing prevalence in human carotid plaques with respect to plaque phenotype to determine the value of hybrid imaging for the detection of these biomarkers.

**Methods:**

Human carotid plaque segments, obtained from endarterectomy, were incubated in [111In]In-DOTA-butylamino-NorBIRT ([111In]In-Danbirt), targeting Leukocyte Function-associated Antigen-1 (LFA-1) on leukocytes. By performing SPECT/CT, both inflammation from DANBIRT uptake and calcification from CT imaging were assessed. Plaque phenotype was classified using histology.

**Results:**

On a total plaque level, comparable levels of calcification volume existed with different degrees of inflammation and vice versa. On a segment level, an inverse relationship between calcification volume and inflammation was evident in highly calcified segments, which classify as fibrocalcific, stable plaque segments. In contrast, segments with little or no calcification presented with a moderate to high degree of inflammation, often coinciding with the more dangerous fibrous cap atheroma phenotype.

**Conclusion:**

Calcification imaging alone can only accurately identify highly calcified, stable, fibrocalcific plaques. To identify high-risk plaques, with little or no calcification, hybrid imaging of calcification and inflammation could provide diagnostic benefit.

**Supplementary Information:**

The online version contains supplementary material available at 10.1007/s12350-021-02745-0.

## Introduction

Atherosclerosis is a chronic, lipid-driven inflammatory disease of the arteries, characterized by the formation of atherosclerotic plaques. The rupture of a plaque in the carotid artery and subsequent thrombus formation and embolization can result in ischemic stroke. Not all plaques rupture and the risk of rupture is not solely dependent on plaque size or degree of lumen stenosis.^1^,^2^ Nevertheless, clinical decision-making is mainly based on the assessment of degree of lumen stenosis.^3^ As only a relative minority of asymptomatic patients actually benefit from surgery (carotid endarterectomy),^4^ improved rupture risk assessment determining which patients need invasive therapy is critical. Rupture risk assessment would allow for targeted treatment of a plaque and could reduce the risk of potentially life-threatening cardiovascular events. Equally important, it would identify the patient not at risk and therefore reduce the number of unnecessary, risky, and costly interventions.^5^ When plaque composition was shown to be a critical determinant of rupture risk and more important than plaque size and luminal stenosis,^1^ the search for non-invasive imaging methods that can specifically identify plaque components became of clinical interest.

Among others, both inflammation and calcification are plaque compositional characteristics that have been linked to stroke risk.^6^–^8^ Inflammation is a hallmark of atherosclerosis and plays a role in all stages of the disease. Inflammation and calcification are intricately connected as inflammation can induce osteogenic transition of vascular smooth muscle cells and inflammatory cells themselves can release calcifying extracellular vesicles,^9^,^10^ while, vice versa, early (micro) calcification appears to induce further inflammation.^11^ It is generally believed that calcification follows inflammation and that advanced calcification is concomitant with a reduction in inflammation,^12^ presenting a stable end-stage of the disease. However, contrary to that notion, specific patterns of advanced calcifications, as visible on CT, play a role in plaque vulnerability,^13^–^15^ including spotty calcifications that were linked to carotid vulnerable plaque phenotype.^16^–^21^

Single photon emission computed tomography (SPECT) and positron emission tomography (PET) have the unique capability to target specific biological processes like inflammation and are used in combination with multi-slice computed tomography (CT) which can visualize the volume and pattern of calcification. Atherosclerotic inflammation is driven by multiple key leukocyte subsets including the monocyte-derived macrophages, lymphocytes, and neutrophils.^22^,^23^ Therefore, it is important to detect the presence of all leukocyte subsets in order to encapsulate the total inflammation activity.^24^ Numerous radiotracers, capable of targeting inflammatory cell subsets, have been developed and were tested for imaging human atherosclerotic plaques.^25^ We recently demonstrated the value of targeting Leukocyte Function-associated Antigen-1 (LFA-1), which is expressed by all leukocytes, with the radiotracer [111In]In-DOTA-butylamino-NorBIRT, also known as [111In]In-DANBIRT, for atherosclerotic plaque imaging with SPECT/CT as a candidate for total inflammation imaging and plaque phenotype classification.^26^–^28^ In these studies, we demonstrated co-localization of [111In]In-DANBIRT uptake with LFA-1-expressing cells, and co-localization of LFA-1-expressing cells with cells expressing markers of activated macrophages.

With hybrid SPECT/CT imaging, we investigated the relation between calcification features, including location and morphology, as derived from CT imaging, and degree of inflammation from DANBIRT SPECT imaging, in relation to plaque phenotype, as assessed with histological analysis.

## Methods

### Sample Acquisition

Human carotid plaque samples were obtained from seven patients undergoing carotid endarterectomy (CEA) in the Erasmus MC, University Medical Center Rotterdam, The Netherlands in a manner that conformed to the declaration of Helsinki and was approved by the hospital’s Ethical Research Committee (MEC 2008-147). The carotid plaques were divided into segments of 2 mm thickness (N = 90) for SPECT/CT imaging followed by immunohistochemistry.

### SPECT/CT Imaging

The plaque segments were incubated with a solution of 100 MBq·nmol of the LFA-1 targeting tracer [^111^In]In-DOTA-butylamino-NorBIRT (DANBIRT) (MW = 886.5 g·mol) for 1 hour as previously reported.^27^ Radiochemical purity (> 90%) and incorporation yield (> 99%) of the tracer were determined by high-pressure liquid chromatography. Segments were subsequently imaged using a VECTor5/CT system (MILabs B.V. Utrecht, The Netherlands) equipped with a high-energy ultra-high-resolution mouse (HE-UHR-M) collimator, yielding a 500 μm reconstructed SPECT resolution and a SPECT sensitivity of ~ 0.3%.^29^,^30^ Each scan was acquired in list mode using the following scan parameters: spiral trajectory bed movement,^31^ in fine step mode at 30 seconds per bed position yielding a total acquisition time of 90 minutes for a field of view of 26 mm in diameter and 48 mm in length. In a single scan, six 2-mm plaque segments, placed in the sample holder, were scanned. Two photopeak windows, incorporating a width of 20 % of the gamma photopeak at 171 and 245 keV,^32^ were set. The triple-energy window method^33^ was applied to each photopeak for scatter correction. Using the Similarity-Regulated Ordered Subset Expectation Maximization (SROSEM) algorithm,^31^ SPECT scans were reconstructed at 0.2 mm voxel size, resulting in 9 iterations, 128 subsets, and a 3D Gaussian post filter of 0.5 mm (FWHM). The VECTor5/CT system was calibrated to a standard of known activity, measured in a dose calibrator, for absolute SPECT quantification.

CT scanner settings were identical for all scans. Ultra-focus magnification with a full scan angle, at 0.24 mA, 50 kV, and 75 ms was applied, yielding a total scan time of 15 minutes. CT scan reconstruction was performed using filtered back projection at a resolution of 20 µm, subsequently reconstructions were downsampled to a resolution of 80 µm for registration to SPECT scans. After imaging, the tissue segments were embedded in tissue-tek medium and stored at − 80 °C until the radioactivity contained in the samples had decayed, after which the segments were processed for immunohistological analysis.

### Data Analysis

Analysis of SPECT scans was performed using PMOD (PMOD Technologies LLC, Zürich, Switzerland, Version 3.4). For each 2-mm plaque segment, total DANBIRT uptake was measured per plaque volume (kBq·mm^3^), at increments of 500μm, concomitant to the SPECT resolution.

The co-registered CT scans were analyzed using 3D slicer. On each CT slice, the plaque tissue was segmented using a semi-automatic level tracing algorithm (opensource software package 3D Slicer version 4.10, www.slicer.org)^34^ which segmented calcification from the plaque tissue at a threshold of > 1600 Hounsfield units. The percent calcification volume fraction was determined as the ratio of calcification volume to total plaque volume.

### Calcification and Inflammation Classification

The plaque segments were classified according to the calcification patterns identified on CT, blinded to the results of DANBIRT SPECT inflammation imaging. **None**: whereby no CT-detectable calcification was present. **Sheet-like (outer border)**: a mixture of speckled and fragmented calcification oriented circumferentially along the outer border of plaque (as described for coronary plaques by^35^) and **Sheet-like (superficial)**: at the lumen border. **Speckled/Spotty**: small flecks of calcification particles or single spotty inclusions. **Diffuse**: cluster of calcification fragments or continuous calcification located in close proximity to the lumen.^35^,^36^ For typical examples see supplemental figure S1. The plaque segments were classified according to the degree of inflammation, as obtained from DANBIRT SPECT, blinded to the results of CT calcification imaging. The degree of inflammation was, based on our dataset, distributed in tertiles: **Low** ≤ 14.4 kBq·mm^3^, **Moderate** 14.4 < kBq·mm^3^ < 19.5, and **High** ≥ 19.5 kBq·mm^3^. For typical examples see supplemental figure S2.

### Plaque Phenotypic Classification

After the radioactivity decayed, each 2-mm plaque segment was cryo-sectioned, at 500 μm increments in line with the SPECT resolution, for quantification and analysis with respect to the SPECT/CT scan data. Within each 500 μm block, sequential adjacent sections of 5 μm were made and stained with Hematoxylin and Eosin to determine the plaques overall structural morphology or with anti-LFA-1 (mouse anti-human CD11a 1:100 Biorad, MCA1848 clone 38) to determine the presence of inflammatory cells (leukocytes). The 90 carotid plaque segments were classified into 3 groups according to the American Heart Association (AHA) plaque classification,^37^ based on these distinct morphological characteristics, blinded to the results of CT calcification and DANBIRT SPECT inflammation imaging. Segments with pathological intimal thickening (PIT N = 15, 17%) contained areas of inflammatory infiltration without necrotic tissue. The fibrous cap atheroma segments (FCA N = 42, 47%) morphologically consisted of a well-formed necrotic core and an overlying fibrous cap containing a high infiltration of inflammatory cells. The fibrocalcific segments (FCALC N = 33, 37%) contained large areas of calcification within the necrotic core. For typical examples see supplemental figure S3.

### Statistical Analysis

Statistical analysis was performed using IBM SPSS statistics 21. A Shapiro–Wilk test was performed to assess the distribution of the data and select the most appropriate statistical test. Spearman’s rho correlation (*r*_s_) was used to compare the correlation between Calcification Volume Fraction (%) and total DANBIRT uptake_mean_.

## Results

### Correlation Between Total Plaque Calcification and Inflammation

The total calcification volume fraction and mean total DANBIRT uptake values for each CEA sample are reported in Table 1. Each CEA sample exhibited a large degree of heterogeneity in terms of inflammation and calcification. Two CEA samples with similar total calcification volume fractions, e.g., nr. 3 and 7, displayed the highest and lowest total DANBIRT uptake_mean_. Conversely, two CEA samples with similar, moderate, total DANBIRT uptake_mean_, e.g., nr. 1 and 4, displayed the highest and lowest total calcification volume fraction.

**Table 1:** Total calcification volume fraction and mean total DANBIRT uptake values on a total plaque level

Plaque (N = segments)	1 (N = 15)	2 (N = 15)	3 (N = 4)	4 (N = 8)	5 (N = 12)	6 (N = 20)	7 (N = 16)
Total % CVF	1.3	5.6	9.7	15.6	4.3	2.9	8.5
Total Uptake_Mean_ (kBq.mm3)	16.6 ± 2.8	16.8 ± 5.9	11.0 ± 2.0	16.8 ± 3.4	18.0 ± 3.7	16.6 ± 6.6	20.1 ± 8.9

### Correlation Between Calcification and Inflammation on a Cross-Sectional Level

**Figure 1:**
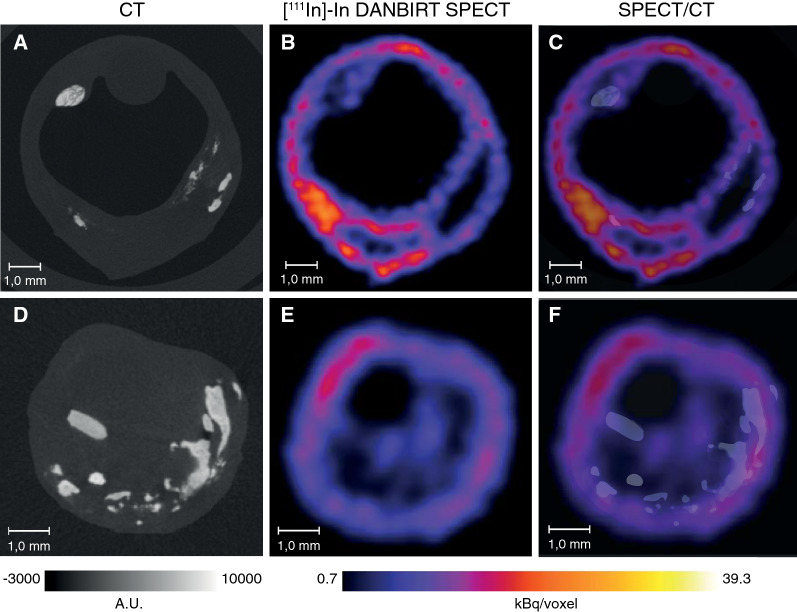
CT images (**A**, **D**) and corresponding SPECT images of uptake for [^111^In]In-DANBIRT (**B**, **E**) with overlay image (**C**, **F**) of two typical examples (1 **A**-**C**, 2 **D**-**F**). *A.U*, arbitrary units

**Figure 2:**
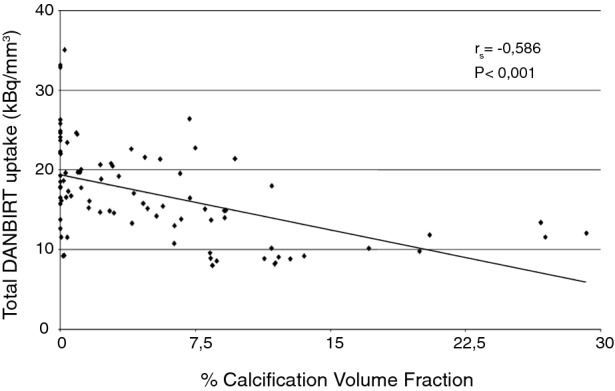
Correlation between percentage calcification volume fraction and total DANBIRT uptake values for each 2-mm segment analyzed

The degree of heterogeneity present within a whole plaque is also evident on the level of the 2-mm plaque segments. Inflammation co-exists with calcifications on a cross-sectional level, although the regions of focal peak DANBIRT uptake are generally spatially distinct from the calcifications (Figure 1). Figure 2 represents the calcification volume fraction and total DANBIRT uptake values for each 2-mm segment of the 7 CEA samples (in total N = 90 segments). In the segments with a calcification volume fraction below 10%, particularly in the segments without any calcification, a large variation in inflammation is evident. However, in segments with a large calcification volume fraction (> 10%), inflammation is low. Overall, a significant inverse relation between calcification and inflammation is seen.

### Correlation Between Calcification Pattern, Degree of Inflammation, and Plaque Phenotype

High-resolution DANBIRT SPECT/CT imaging reveals the spatial interplay between calcification and focal regions of DANBIRT uptake, representing areas of leukocyte infiltration. The plaque segments were classified according to the calcification patterns identified on CT: none (N = 20, 22%), sheet-like (outer border) (N = 11, 12%), sheet-like (superficial) (N = 18, 20%), speckled/ Spotty (N = 20, 22%), and diffuse (N = 21, 23%). Plaque phenotype was classified as pathological intimal thickening (PIT) (N = 15, 17%), fibrous cap atheroma (FCA) (N = 38, 42%), or fibrocalcific plaque (FCALC) (N = 37, 41%). The analysis of each 2-mm plaque segment in terms of degree of inflammation, with respect to plaque phenotype, for each given calcification pattern is presented in Table S1 in the supplement.

**Figure 3:**
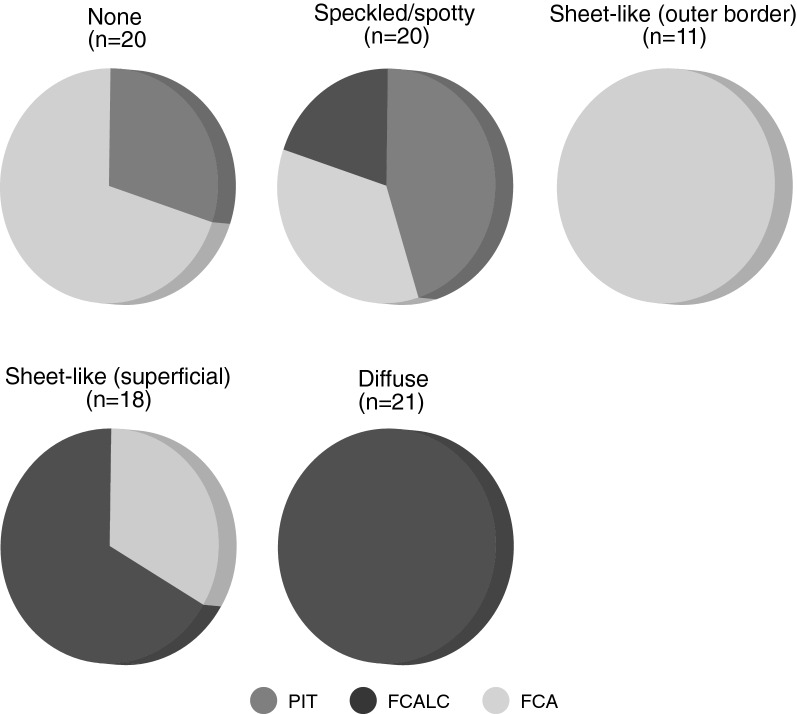
Calcification pattern with the accompanying plaque phenotypes for each 2-mm segment analyzed (*PIT*, pathological intimal thickening; *FCALC*, fibrocalcific; *FCA*, fibrous cap atheroma)

**Figure 4:**
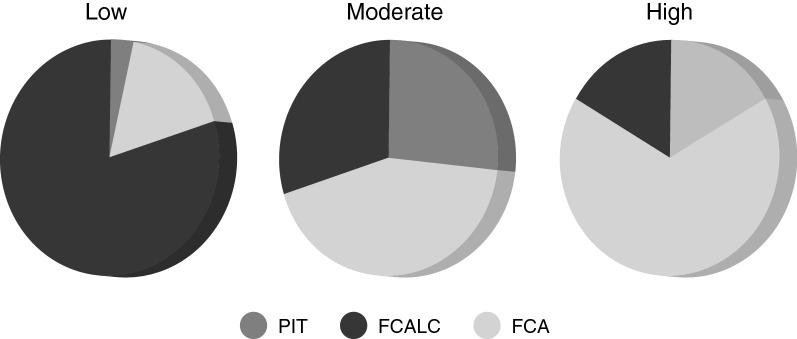
Degree of [^111^In]In-DANBIRT uptake with the accompanying plaque phenotypes for each 2-mm segment analyzed (*PIT*, pathological intimal thickening; *FCALC*, fibrocalcific; *FCA*, fibrous cap atheroma)

**Figure 5:**
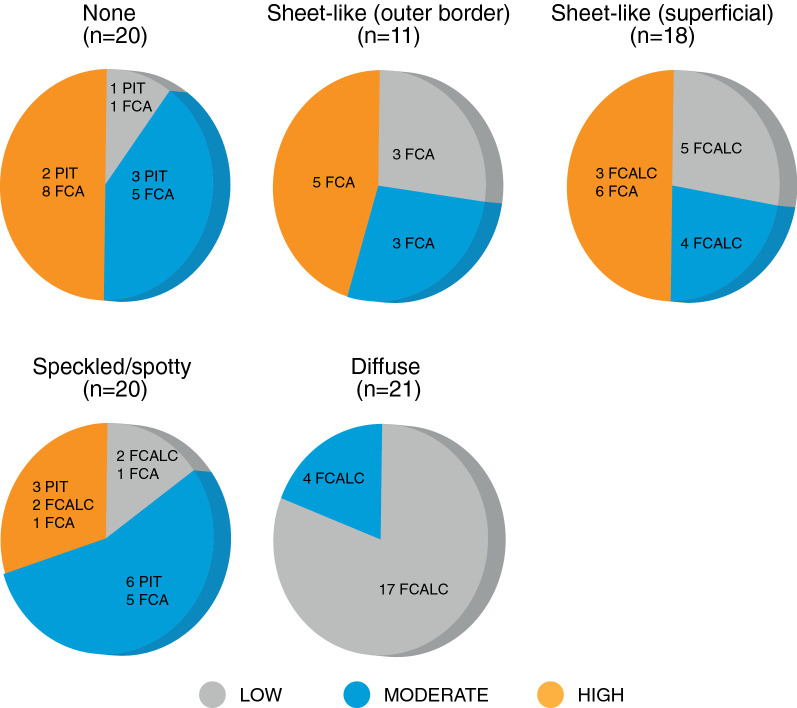
Calcification pattern with the accompanying degree of [^111^In]In-DANBIRT uptake and plaque phenotypes for each 2-mm segment analyzed (*PIT*, pathological intimal thickening; *FCALC*, fibrocalcific; *FCA*, fibrous cap atheroma)

The analysis of calcification pattern in each 2-mm plaque segment with respect to a distinct plaque phenotype (Figure 3) revealed that, all segments with diffuse calcification on CT imaging classify as fibrocalcific plaques, while all segments with sheet-like outer border calcification classify as fibrous cap atheroma. The segments with speckled/ spotty calcification or sheet-like superficial calcification are not linked to a particular plaque phenotype. 14/20 segments without any calcification classify as fibrous cap atheroma. Quantification of inflammation, represented by DANBIRT uptake, in each 2-mm plaque segment with distinct plaque phenotype (Figure 4) revealed that most segments with low inflammation classify as fibrocalcific plaques (24/30). Moderate and high inflammation can be present in all plaque phenotypes, but mostly in fibrous cap atheroma (moderate 13/30, high 20/30). The analysis of calcification pattern and inflammation level in each 2-mm plaque segment with distinct plaque phenotype is depicted in Figure 5. Half of the segments without calcification (“none”) show high inflammation (10/20). 8 of those segments are fibrous cap atheroma. Moderate inflammation is seen in 8/20 with 5 of those segments being fibrous cap atheroma. 1 of 2 segments without calcification and with low inflammation classifies as fibrous cap atheroma. Segments with sheet-like outer border calcification vary in their degree of inflammation but all classify as fibrous cap atheroma. Segments with sheet-like superficial calcification have variable degrees of inflammation. In the case of low or moderate inflammation they all classify as fibrocalcific plaques, but in the case of high inflammation 6/9 classify as fibrous cap atheroma, 3/9 as fibrocalcific. Segments with speckled/ spotty calcification vary in their degree of inflammation. In case of low inflammation, 1/3 classify as fibrous cap atheroma, while the other 2 are classified as fibrocalcific plaques. In the case of moderate inflammation, 6/11 classify as pathological intimal thickening and 5/11 classify as fibrous cap atheroma. In the case of high inflammation, 3/6 classify as pathological intimal thickening, 1/6 as fibrous cap atheroma, and 2/6 as fibrocalcific plaque. All segments with diffuse calcification show low to moderate inflammation and classify as fibrocalcific plaques.

## Discussion

In this study, the co-existing prevalence between inflammation and calcification patterns in human carotid plaques, acquired from carotid endarterectomy procedures, was examined with respect to histologically determined plaque phenotype. By performing [^111^In]In-DANBIRT SPECT/CT on CEA samples, both imaging biomarkers were assessed ex vivo on a total plaque level and on a cross-sectional level.

The relationship between inflammation and calcification was diverse. On a total plaque level, our advanced multi-modal imaging approach reveals large variability in the presence of these imaging biomarkers. Comparable levels of calcification volume can present with different degrees of inflammation and vice versa. On a segment level, the previously reported^38^ inverse relationship between calcification volume and inflammation is evident, mainly in highly calcified plaques. However, plaque segments with little or no calcification, can vary greatly in their degree of inflammation. These findings are in line with the theory that inflammation precedes calcification.^12^ However, even though regions of focal peak uptake of DANBIRT were spatially distinct from the calcified areas, confirming the inverse relationship, areas with extensive inflammation do co-exist with calcifications on a cross-sectional level. These findings are in line with the findings from Joshi et al.^11^ They used [^18^F]FDG-PET to longitudinally assess plaque inflammation and CT for calculation of plaque calcification and reported that plaques with progressive calcification from baseline to follow-up, showed a high degree of inflammation. These findings would indicate that inflammation is still active after calcification has occurred. This inflammation can affect vulnerability as is evident by our finding of a high number of fibrous cap atheroma classified segments with a high degree of inflammation as determined by DANBIRT uptake (Figure 4 and^27^).

Specific features of calcifications have been linked to carotid vulnerable plaque phenotype. In both coronary^17^,^18^,^39^,^40^ and carotid plaque^16^–^21^, spotty calcifications were associated with high-risk vulnerable plaques. In our study, we classified the calcification patterns in the plaque segments as sheet-like (outer border or superficial), spotty, or diffuse. All segments with diffuse calcification were classified as fibrocalcific phenotypes (Type VII Virmani AHA), representing an advanced, but stable, phase of the disease. These segments generally associated with a low degree of inflammation. Segments with sheet-like calcifications, located at the outer border, were all classified as fibrous cap atheroma (Type V Virmani AHA), representing a more dangerous plaque phenotype. During the calcification process, calcifications start to develop at the intima/media interface. These sheets of calcification might be stabilizing from a mechanical perspective, but are still covered by soft necrotic tissue in the ‘early’ phases of this process. This in combination with (moderate to high) inflammation, which is known to weaken the plaque/cap by enzymatic collagen degradation,^41^ could potentially increase the risk of rupture, underscoring the added value of imaging both calcification and inflammation. This is also the case for plaques not showing any calcification on CT. Most of the non-calcified segments in our study show a moderate to high degree of inflammation, which in many cases coincides with a more dangerous plaque segment phenotype, underlining that non-calcified plaques might benefit even more from inflammation imaging.

The remaining calcification patterns, i.e., spotty and sheet-like superficial, did not show a correlation to a particular phenotype. Inflammation was variable in these segments and did not aid in the identification of the more dangerous fibrous cap atheroma. Although some of the segments with spotty calcification were classified as fibrous cap atheroma, we could not confirm the previously reported link^16^–^21^ between spotty calcifications and high-risk plaque in our dataset.

There are inherent limitations associated with this study that must be taken into account. Our findings are based on a small sample size and an *ex vivo* analysis. The clinical translational aspect of calcification pattern and inflammation imaging needs to be assessed on a larger scale *in vivo*, specifically regarding differences in clinical and pre-clinical imaging resolution. PET scanning is currently the preferred methodology in atherosclerosis imaging due to the superior spatial resolution and quantification capabilities compared with clinical SPECT. PET imaging can be performed with DANBIRT as it can be labeled with PET radioisotopes^42^ but clinical PET resolution is approximately 10 times lower^43^ than the pre-clinical SPECT resolution of 0.5 mm used in this study. Irrespective of the nuclear imaging technique applied, the fundamental principle of imaging leukocytes in atherosclerotic plaque tissue remains the same and the differences in DANBIRT uptake between plaque phenotypes are expected to be comparable in an in vivo setting.

## Conclusion

Calcification imaging alone can only accurately identify highly calcified, stable, fibrocalcific plaques. Calcification and inflammation can be assessed simultaneously in vivo by combining nuclear imaging (e.g., [^18^F]FDG-PET) with CT. To identify high-risk, vulnerable plaques, with little or no calcification, it is interesting to explore the additive value of these biomarkers to noninvasively identify vulnerable carotid plaque.

## New Knowledge Gained

In carotid atherosclerotic plaques, comparable levels of calcification volume can exist with different degrees of inflammation and vice versa. The previously reported inverse relationship between calcification volume and inflammation is only evident in highly calcified segments, which classify as fibrocalcific, stable plaque segments. However, segments with little or no calcification can present with a moderate to high degree of inflammation and often classify as a more dangerous fibrous cap atheroma phenotype. In this regard, hybrid PET/CT imaging of both inflammation and calcification could provide added diagnostic benefit for the assessment of plaques which could be at risk and identify the patients that might benefit from therapeutic intervention.

## Supplementary Information

Below is the link to the electronic supplementary material.

Supplementary file1 (PPTX 1273 kb)

Supplementary file2 (M4A 10311 kb)

Supplementary file3 (DOCX 1887 kb)

## Abbreviations

LFA-1: Leukocyte function-associated antigen-1
SPECT: Single photon emission tomography
PET: Positron emission tomography
CT: Computed tomography
CEA: Carotid endarterectomy
PIT: Pathological intimal thickening
FCA: Fibrous cap atheroma
FCALC: Fibrocalcific
