# Supplementary material for: SPECT/CT imaging of inflammation and calcification in human carotid atherosclerosis to identify the plaque at risk of rupture
Source: J Nucl Cardiol. 2021 Jul 27;29(5):2487–96. doi: 10.1007/s12350-021-02745-0 (PMC9553768; doi:10.1007/s12350-021-02745-0)
Supplement: Supplementary file 1 — Supplementary file1 (PPTX 1273 kb) [file 12350_2021_2745_MOESM1_ESM.pptx]

## Slide 1
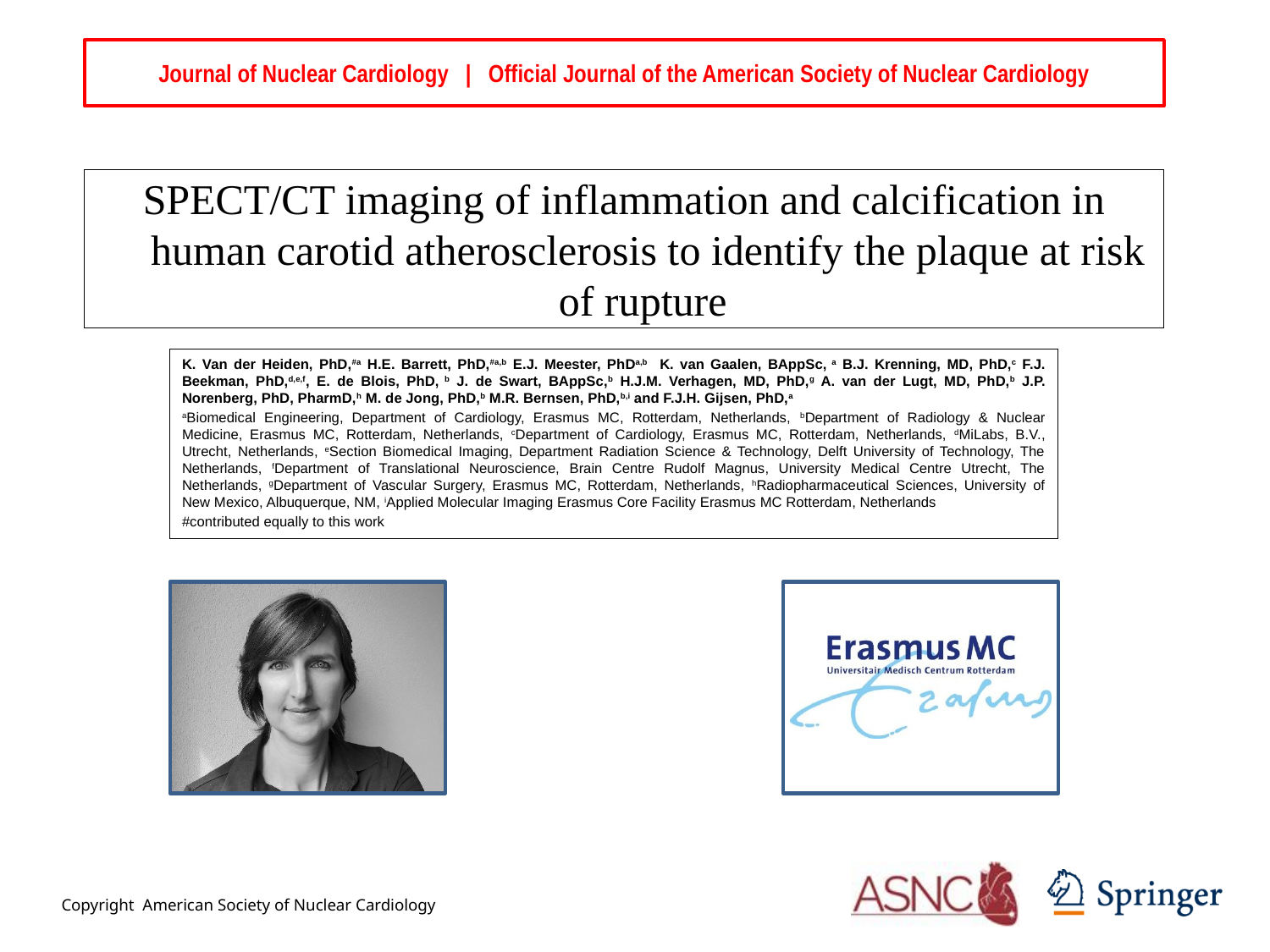

Journal of Nuclear Cardiology | Official Journal of the American Society of Nuclear Cardiology
# SPECT/CT imaging of inflammation and calcification in human carotid atherosclerosis to identify the plaque at risk of rupture
K. Van der Heiden, PhD,#a H.E. Barrett, PhD,#a,b E.J. Meester, PhDa,b K. van Gaalen, BAppSc, a B.J. Krenning, MD, PhD,c F.J. Beekman, PhD,d,e,f, E. de Blois, PhD, b J. de Swart, BAppSc,b H.J.M. Verhagen, MD, PhD,g A. van der Lugt, MD, PhD,b J.P. Norenberg, PhD, PharmD,h M. de Jong, PhD,b M.R. Bernsen, PhD,b,i and F.J.H. Gijsen, PhD,a
aBiomedical Engineering, Department of Cardiology, Erasmus MC, Rotterdam, Netherlands, bDepartment of Radiology & Nuclear Medicine, Erasmus MC, Rotterdam, Netherlands, cDepartment of Cardiology, Erasmus MC, Rotterdam, Netherlands, dMiLabs, B.V., Utrecht, Netherlands, eSection Biomedical Imaging, Department Radiation Science & Technology, Delft University of Technology, The Netherlands, fDepartment of Translational Neuroscience, Brain Centre Rudolf Magnus, University Medical Centre Utrecht, The Netherlands, gDepartment of Vascular Surgery, Erasmus MC, Rotterdam, Netherlands, hRadiopharmaceutical Sciences, University of New Mexico, Albuquerque, NM, iApplied Molecular Imaging Erasmus Core Facility Erasmus MC Rotterdam, Netherlands
#contributed equally to this work
Copyright American Society of Nuclear Cardiology

## Slide 2
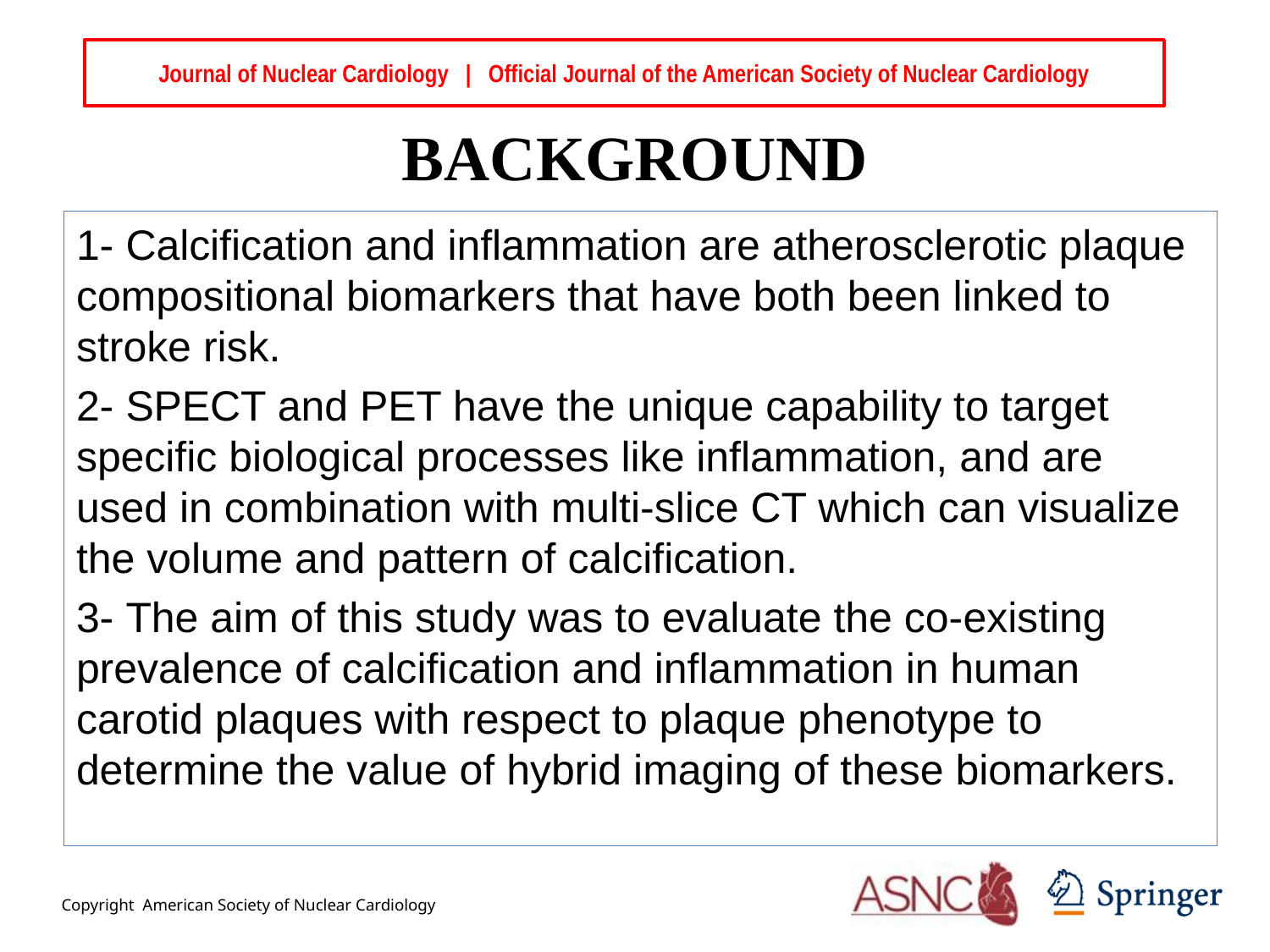

Journal of Nuclear Cardiology | Official Journal of the American Society of Nuclear Cardiology
# BACKGROUND
1- Calcification and inflammation are atherosclerotic plaque compositional biomarkers that have both been linked to stroke risk.
2- SPECT and PET have the unique capability to target specific biological processes like inflammation, and are used in combination with multi-slice CT which can visualize the volume and pattern of calcification.
3- The aim of this study was to evaluate the co-existing prevalence of calcification and inflammation in human carotid plaques with respect to plaque phenotype to determine the value of hybrid imaging of these biomarkers.
Copyright American Society of Nuclear Cardiology

## Slide 3
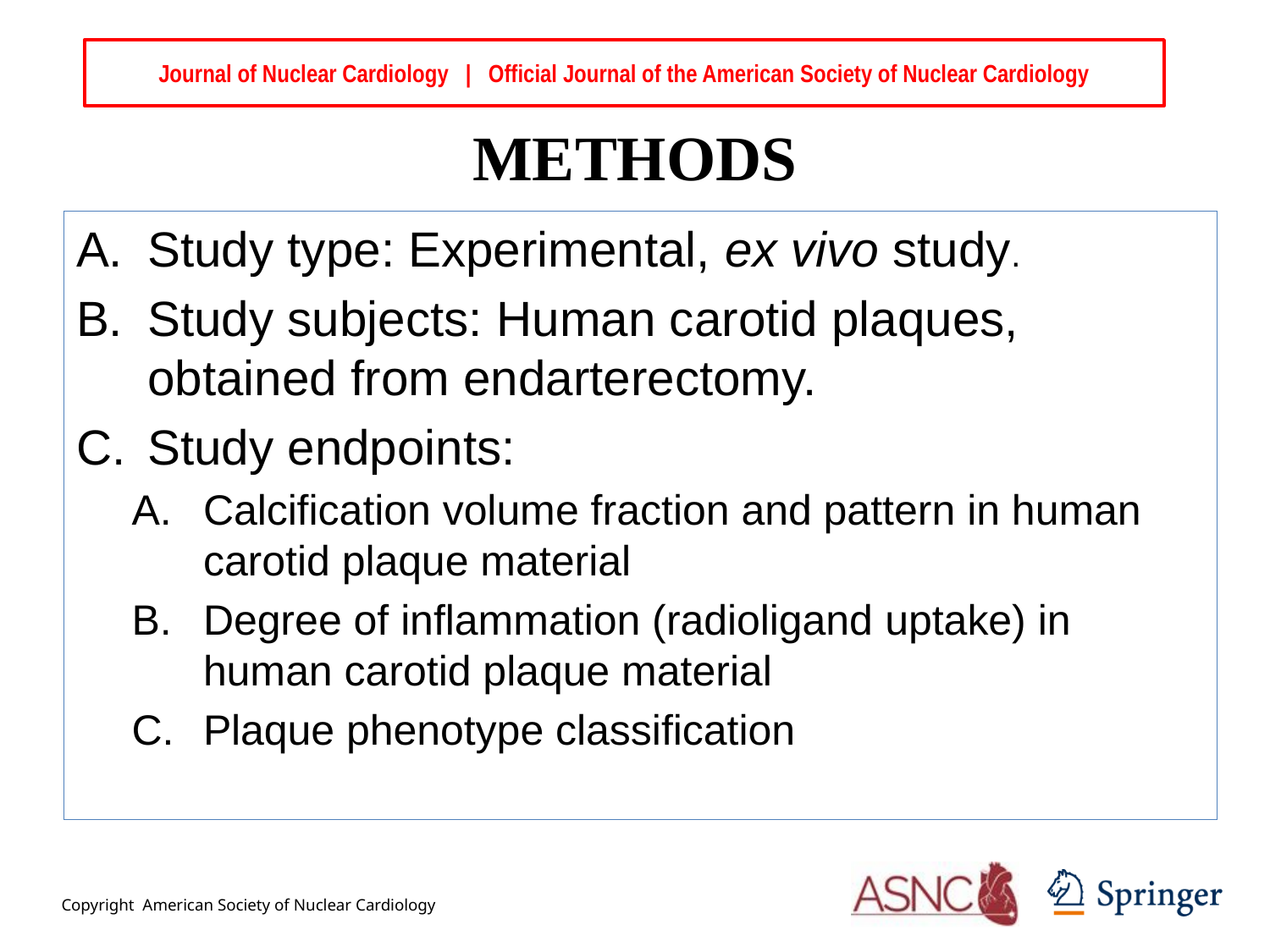

Journal of Nuclear Cardiology | Official Journal of the American Society of Nuclear Cardiology
# METHODS
Study type: Experimental, ex vivo study.
Study subjects: Human carotid plaques, obtained from endarterectomy.
Study endpoints:
Calcification volume fraction and pattern in human carotid plaque material
Degree of inflammation (radioligand uptake) in human carotid plaque material
Plaque phenotype classification
Copyright American Society of Nuclear Cardiology

## Slide 4
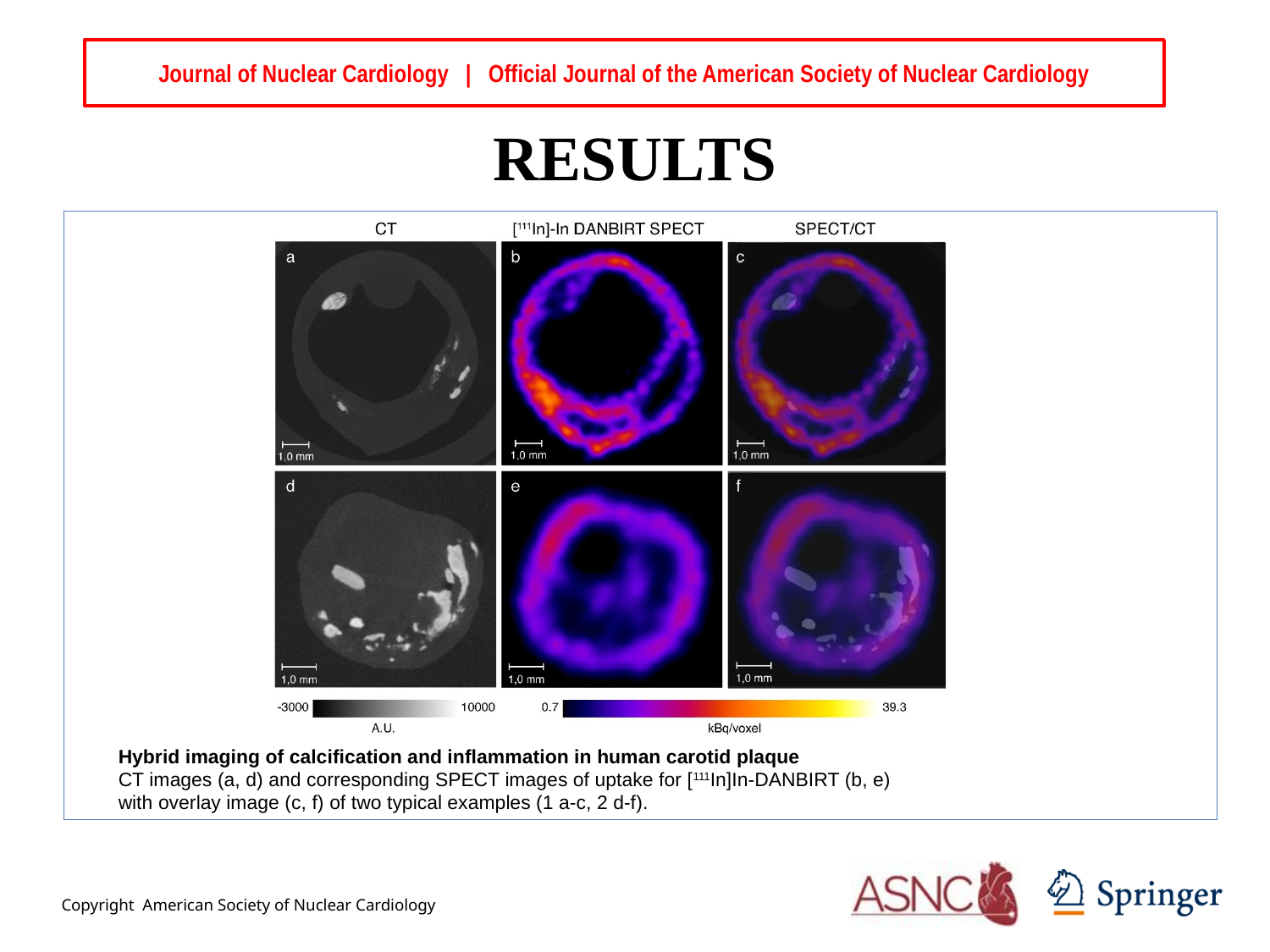

Journal of Nuclear Cardiology | Official Journal of the American Society of Nuclear Cardiology
# RESULTS
Hybrid imaging of calcification and inflammation in human carotid plaque
CT images (a, d) and corresponding SPECT images of uptake for [111In]In-DANBIRT (b, e)
with overlay image (c, f) of two typical examples (1 a-c, 2 d-f).
Copyright American Society of Nuclear Cardiology

## Slide 5
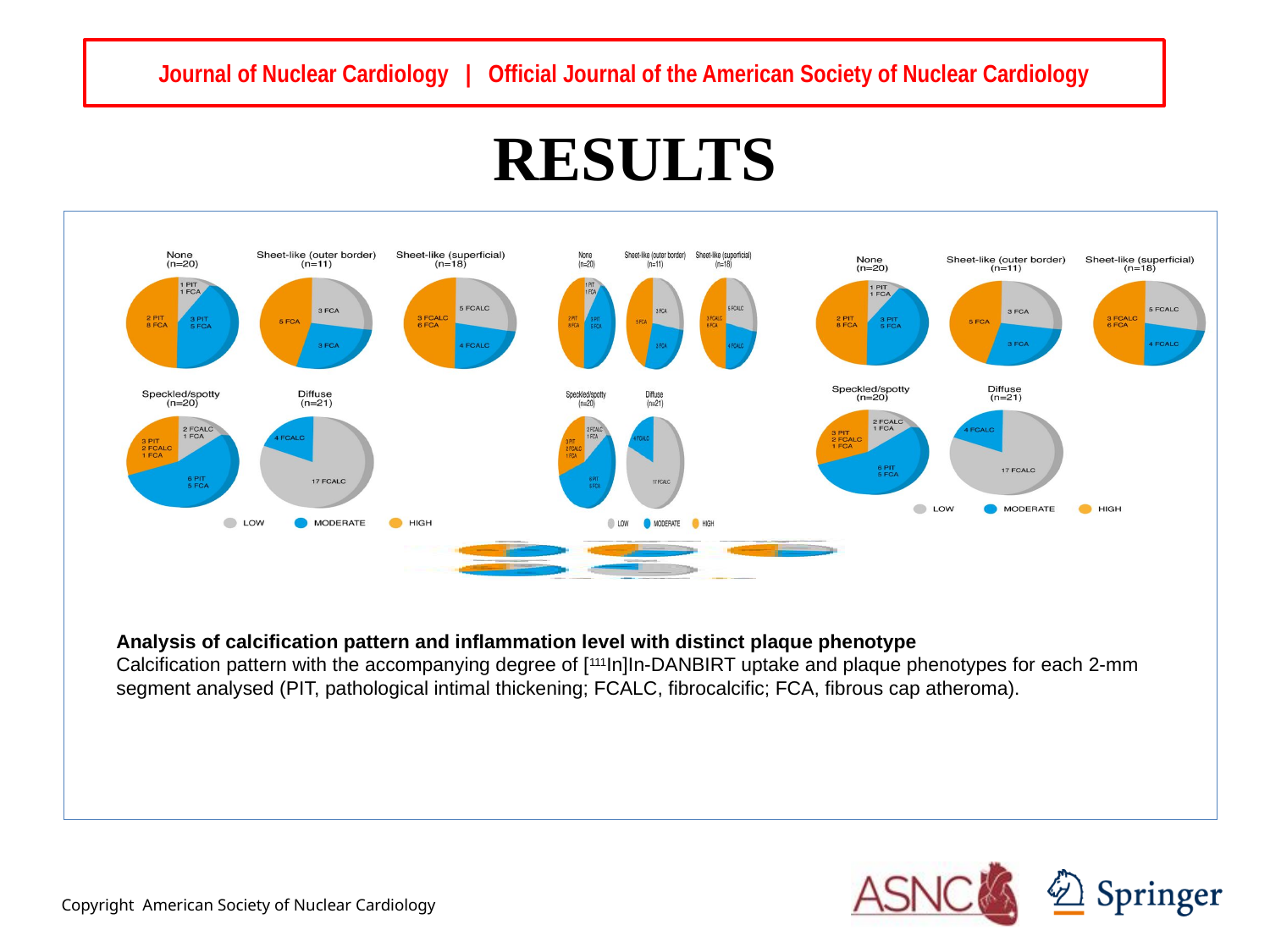

Journal of Nuclear Cardiology | Official Journal of the American Society of Nuclear Cardiology
# RESULTS
Analysis of calcification pattern and inflammation level with distinct plaque phenotype
Calcification pattern with the accompanying degree of [111In]In-DANBIRT uptake and plaque phenotypes for each 2-mm segment analysed (PIT, pathological intimal thickening; FCALC, fibrocalcific; FCA, fibrous cap atheroma).
Copyright American Society of Nuclear Cardiology

## Slide 6
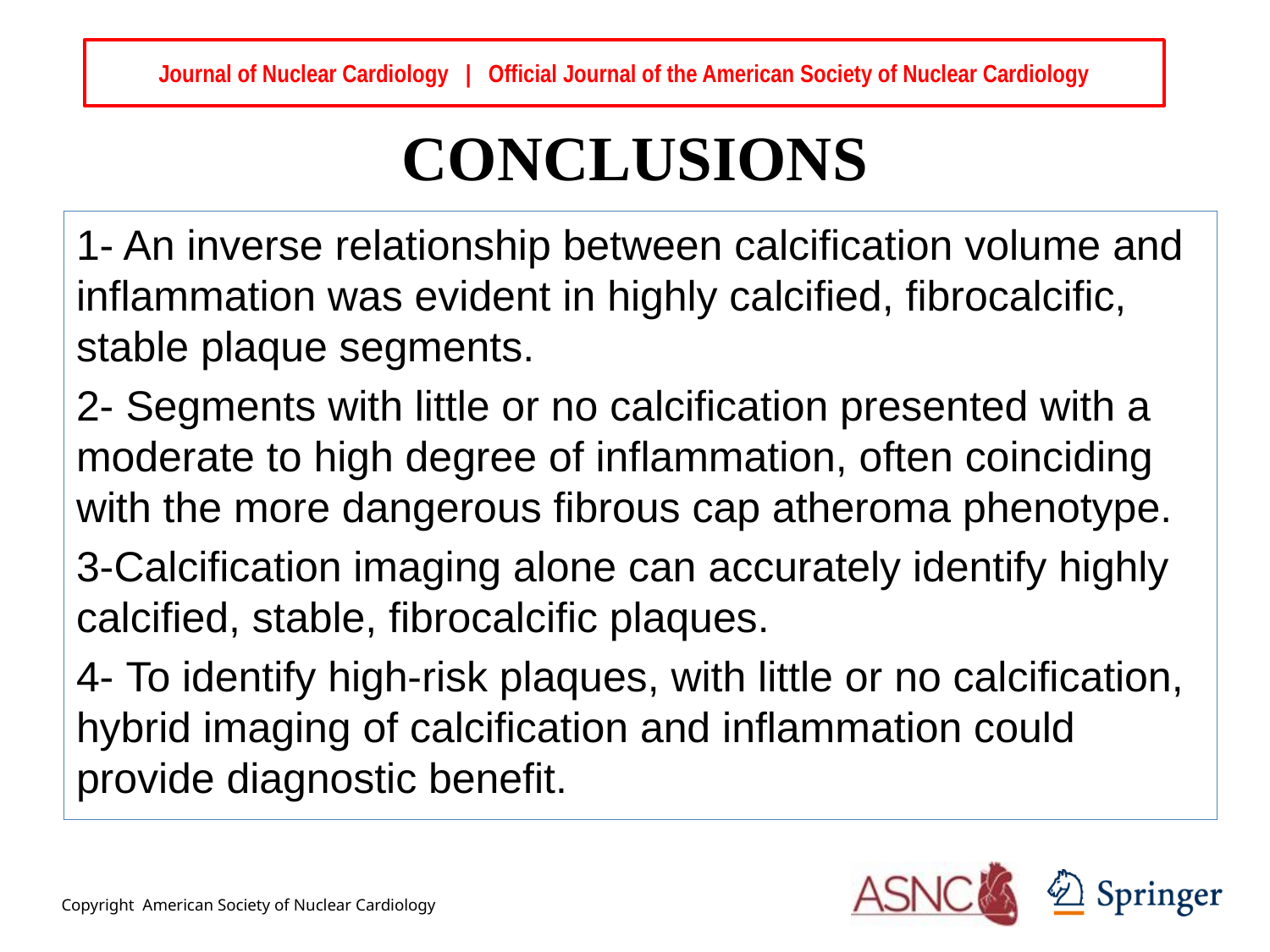

Journal of Nuclear Cardiology | Official Journal of the American Society of Nuclear Cardiology
# CONCLUSIONS
1- An inverse relationship between calcification volume and inflammation was evident in highly calcified, fibrocalcific, stable plaque segments.
2- Segments with little or no calcification presented with a moderate to high degree of inflammation, often coinciding with the more dangerous fibrous cap atheroma phenotype.
3-Calcification imaging alone can accurately identify highly calcified, stable, fibrocalcific plaques.
4- To identify high-risk plaques, with little or no calcification, hybrid imaging of calcification and inflammation could provide diagnostic benefit.
Copyright American Society of Nuclear Cardiology
