# Supplementary material for: SPECT/CT imaging of inflammation and calcification in human carotid atherosclerosis to identify the plaque at risk of rupture
Source: J Nucl Cardiol. 2021 Jul 27;29(5):2487–96. doi: 10.1007/s12350-021-02745-0 (PMC9553768; doi:10.1007/s12350-021-02745-0)
Supplement: Supplementary file 3 — Supplementary file3 (DOCX 1887 kb) [file 12350_2021_2745_MOESM3_ESM.docx]

**SUPPLEMENT**

**SPECT/CT imaging of inflammation and calcification in human carotid atherosclerosis to identify the plaque at risk of rupture**

**K. Van der Heiden, PhD,^#a^ H.E. Barrett, PhD,^#a,b^ E.J. Meester, PhD^a,b^ K. van Gaalen, BAppSc, ^a^ B.J. Krenning, MD, PhD,^c^ F.J. Beekman, PhD,^d,e,f^, E. de Blois, PhD, ^b^ J. de Swart, BAppSc,^b^ H.J.M. Verhagen, MD, PhD,^g^ A. van der Lugt, MD, PhD,^b^ J.P. Norenberg, PhD, PharmD,^h^ M. de Jong, PhD,^b^ M.R. Bernsen, PhD,^b,i^ and F.J.H. Gijsen, PhD,^a^**

^a^Biomedical Engineering, Department of Cardiology, Erasmus MC, Rotterdam, Netherlands

^b^Department of Radiology & Nuclear Medicine, Erasmus MC, Rotterdam, Netherlands

^c^Department of Cardiology, Erasmus MC, Rotterdam, Netherlands

^d^MiLabs, B.V., Utrecht, Netherlands

^e^Section Biomedical Imaging, Department Radiation Science & Technology, Delft University of Technology, The Netherlands

^f^Department of Translational Neuroscience, Brain Centre Rudolf Magnus, University Medical Centre Utrecht, The Netherlands

^g^Department of Vascular Surgery, Erasmus MC, Rotterdam, Netherlands

^h^Radiopharmaceutical Sciences, University of New Mexico, Albuquerque, NM

^i^Applied Molecular Imaging Erasmus Core Facility Erasmus MC Rotterdam, Netherlands

#contributed equally to this work

**Corresponding Author:** K. Van der Heiden, PhD, Department of Biomedical Engineering, Thorax Center, Erasmus Medical Center, 3000 CA Rotterdam, The Netherlands; k.vanderheiden@erasmusmc.nl

**Submitted to Journal of Nuclear Cardiology**

**Table S1** The analysis of each 2-mm plaque segment in terms of degree of inflammation, with respect to plaque phenotype, for each given calcification pattern.

| Calcification pattern | Plaque phenotype | Low  inflammation | Moderate  inflammation | High  inflammation | Total |
| --- | --- | --- | --- | --- | --- |
| None | PIT | 1 | 3 | 2 | 6 |
|  | FCA | 1 | 5 | 8 | 14 |
|  | FCALC | 0 | 0 | 0 | 0 |
|  | Total | 2 | 8 | 10 | 20 |
| Sheet-like  Outer border | PIT | 0 | 0 | 0 | 0 |
|  | FCA | 3 | 3 | 5 | 11 |
|  | FCALC | 0 | 0 | 0 | 0 |
|  | Total | 3 | 3 | 5 | 11 |
| Sheet-like  Superficial | PIT | 0 | 0 | 0 | 0 |
|  | FCA | 0 | 0 | 6 | 6 |
|  | FCALC | 5 | 4 | 3 | 12 |
|  | Total | 5 | 4 | 9 | 18 |
| Speckled/ Spotty | PIT | 0 | 6 | 3 | 9 |
|  | FCA | 1 | 5 | 1 | 7 |
|  | FCALC | 2 | 0 | 2 | 4 |
|  | Total | 3 | 11 | 6 | 20 |
| Diffuse | PIT | 0 | 0 | 0 | 0 |
|  | FCA | 0 | 0 | 0 | 0 |
|  | FCALC | 17 | 4 | 0 | 21 |
|  | Total | 17 | 4 | 0 | 21 |
|  | Overall total | 30 | 30 | 30 | 90 |


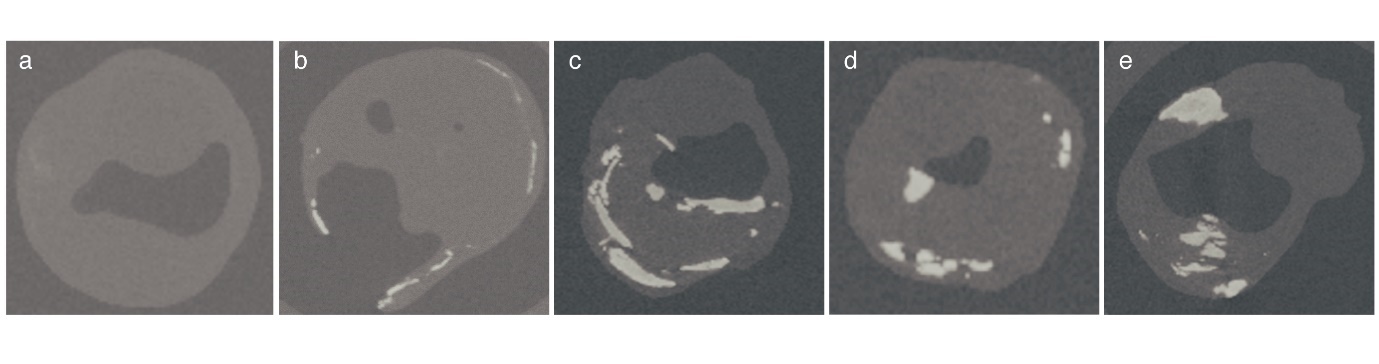


**Fig. S1** Calcification patterns identified on CT. A None, B Sheet-like (outer border), C sheet-like (superficial), D Speckled/ spotty, E Diffuse.

**
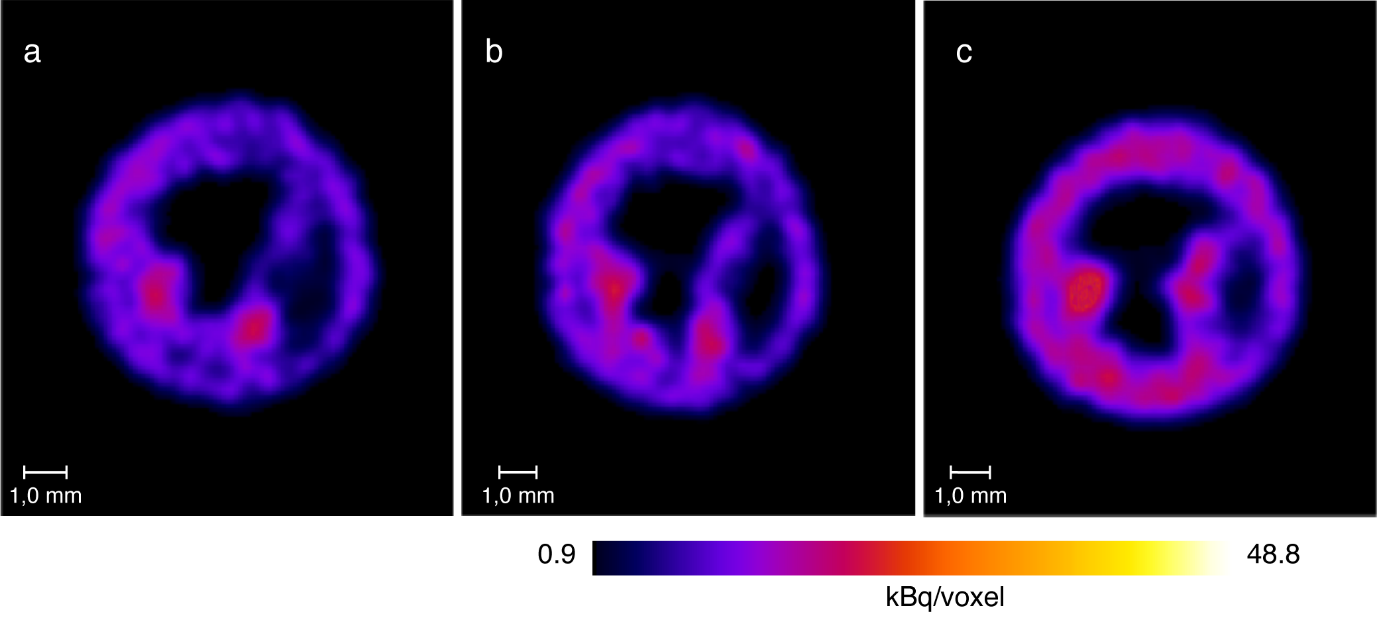
Fig. S2** Degree of inflammation identified by DANBIRT SPECT imaging. a Low, b Moderate, c High.


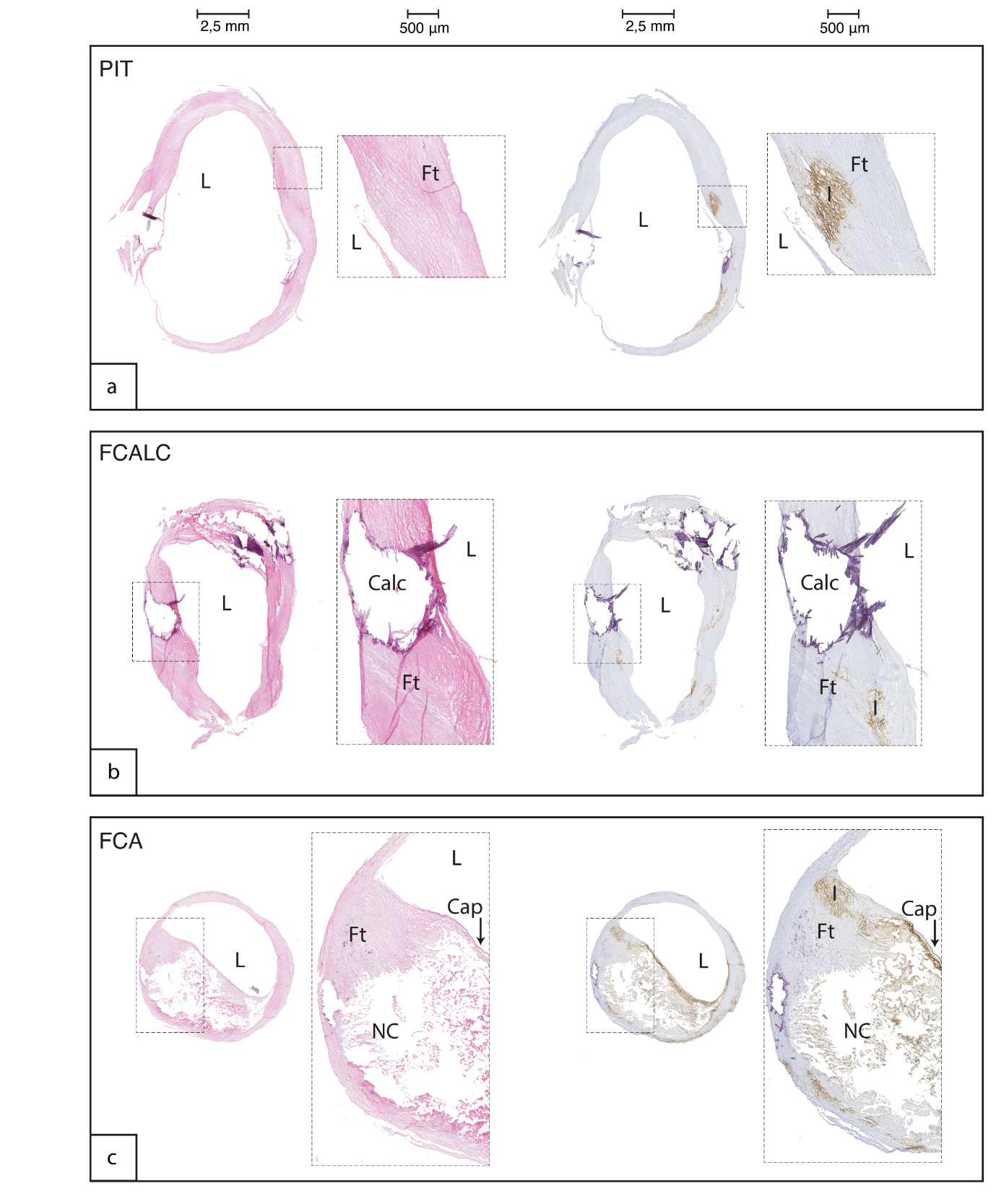


**Fig. S3** Classification plaque phenotype according to the American Heart Association plaque classification based on HE staining (left panel) and LFA-1 staining (right panel), depicting general morphology and inflammation (leukocytes), respectively. The boxed area is shown in higher magnification. a Pathological intimal thickening (PIT), b Fibrocalcific (FCALC), c Fibrous cap atheroma (FCA). L: lumen, Ft: fibrous tissue, I: inflammation, Calc: calcification, NC: necrotic core. Arrow in c magnification depicts the fibrous cap.
